# Factors That Improve RT-QuIC Detection of Prion Seeding Activity

**DOI:** 10.3390/v8050140

**Published:** 2016-05-23

**Authors:** Christina D. Orrú, Andrew G. Hughson, Bradley R. Groveman, Katrina J. Campbell, Kelsie J. Anson, Matteo Manca, Allison Kraus, Byron Caughey

**Affiliations:** Laboratory of Persistent Viral Diseases, Rocky Mountain Laboratories, National Institute for Allergy and Infectious Diseases, National Institutes of Health, Hamilton, 59840 MT, USA; christina.orru@nih.gov (C.D.O.); ahughson@niaid.nih.gov (A.G.H.); bradley.groveman@nih.gov (B.R.G.); katrina.campbell@nih.gov (K.J.C.); kelsiejaness@gmail.com (K.J.A.); matteo.manca@nih.gov (M.M.); allison.kraus@nih.gov (A.K.)

**Keywords:** prion, CJD, olfactory mucosa, RT-QuIC, scrapie, CWD, BSE

## Abstract

Rapid and sensitive detection of prions is important in managing prion diseases. The real-time quaking-induced conversion (RT-QuIC) assay for prion seeding activity has been applied to many prion diseases and provides for specific antemortem diagnostic testing. We evaluated RT-QuIC’s long-term consistency and varied multiple reaction parameters. Repeated assays of a single scrapie sample using multiple plate readers and recombinant prion protein (rPrP^Sen^) substrates gave comparable results. N-terminal truncated hamster rPrP^Sen^ (residues 90–231) hastened both prion-seeded and prion-independent reactions but maintained a clear kinetic distinction between the two. Raising temperatures or shaking speeds accelerated RT-QuIC reactions without compromising specificity. When applied to nasal brushings from Creutzfeldt-Jakob disease patients, higher temperatures accelerated RT-QuIC kinetics, and the use of hamster rPrP^Sen^ (90–231) strengthened RT-QuIC responses. Elongation of shaking periods reduced scrapie-seeded reaction times, but continuous shaking promoted false-positive reactions. Furthermore, pH 7.4 provided for more rapid RT-QuIC reactions than more acidic pHs. Additionally, we show that small variations in the amount of sodium dodecyl sulfate (SDS) significantly impacted the assay. Finally, RT-QuIC performed in multiplate thermoshakers followed by fluorescence readings in separate plate readers enhanced assay throughput economically. Collectively, these results demonstrate improved speed, efficacy and practicality of RT-QuIC assays and highlight variables to be optimized for future applications.

## 1. Introduction

Mammalian prion diseases or transmissible spongiform encephalopathies (TSEs) include Creutzfeldt-Jakob disease (CJD) in humans; scrapie in sheep and goats; bovine spongiform encephalopathy (BSE) in cattle; chronic wasting disease (CWD) in cervids; and various experimental rodent-adapted prion strains. The molecular pathogenesis of TSEs involves the accumulation of abnormal, infectivity-associated forms of the hosts’ prion protein (PrP). While the normal form of PrP, referred to here as PrP^Sen^, is protease-sensitive, mostly monomeric, and rich in α-helices, TSE-associated form(s) (e.g., PrP^Sc^, PrP^CJD^ or PrP^CWD^) tend to be multimeric, relatively protease-resistant, and rich in β-sheet (reviewed in [1]). Here we will refer to protease-resistant forms of PrP as PrP^Res^.

The ability of TSE-associated forms of PrP such as PrP^Res^ to seed the polymerization of recombinant PrP^Sen^ (rPrP^Sen^) into amyloid fibrils that enhance the fluorescence of thioflavin-T (ThT) serves as the basis of sensitive assays to detect prion-associated seeding activity [2,3,4]. One of these assays, the real-time quaking-induced conversion (RT-QuIC) assay, detects prion-seeding activity in a wide variety of tissues and fluids from TSE-infected hosts [3,4]. Coupling of prion capture steps with RT-QuIC (eQuIC) can markedly improve the overall sensitivity of the assay for certain tissues such as plasma [5,6,7,8]. With the 15B3 antibody-based eQuIC, positive reactions can be obtained from as little as 10^14^-fold dilutions of human variant CJD brain tissue containing ~2 ag of PrP^Res^ [5,6]. Without a prion capture step, 10^7^–10^9^-fold dilutions of brain homogenates from a variety of TSE-affected mammals can typically be detected. Quantitation of relative levels of prion seeding activity can be achieved using endpoint dilution RT-QuIC [3] or, under more carefully controlled experimental conditions, comparisons of reaction kinetics [9,10,11].

RT-QuIC tests have been adapted to the detection of many types of prion seeding activity in multiple host species and specimen types including those of particular diagnostic significance such as cerebrospinal fluid (CSF) [4,12,13,14,15], blood [5,6,7], saliva [8] and nasal brushings [16,17]. However, most applications would be aided by improved speed and sensitivity. Thus, we have continued to explore factors that improve the performance and practicality of RT-QuIC assays. We recently reported marked improvements in the speed and sensitivity of detection of human sporadic CJD (sCJD) in CSF based on the selection of substrate, temperature, and sodium dodecyl sulfate (SDS) concentration [18]. Here we report the evaluation of these and other RT-QuIC parameters for the detection of a variety of prion types. Consideration of these findings should facilitate development of applications of RT-QuIC assays for the detection of prions in medicine, agriculture, wildlife biology and research.

## 2. Materials and Methods

### 2.1. Recombinant Prion Protein Purification

We used human (residues 23 to 230; accession no. M13899) and hamster (Syrian golden hamster residues 23–231 and 90–231; accession no. K02234) recombinant prion protein (rPrP^Sen^) that lacked a Histidine tag and were purified as described previously [3] (Table 1). Briefly, *Escherichia coli* (*E. coli*) carrying a pET41 vector (EMD, Billerica, MA, USA) with the PrP sequence was grown in Luria Broth (LB) media in the presence of Kanamycin (0.05 mg/mL) and Chloramphenicol (0.034 mg/mL) and protein expression was induced using Overnight Express Autoinduction System 1 (Novagen, Madison, WI, USA). Inclusion body preparations were isolated from ~4 g of pelleted cells using the Bug Buster Master Mix (Novagen) protocol and stored at −20 °C.

To purify the recombinant prion protein, isolated inclusion bodies were solubilized into 8 M guanidine HCl in 100 mM Na Phosphate, pH 8.0, using an OMNI International TH mixer (OMNI International, Kennesaw, GA, USA) with a disposable tip and incubated with continuous mixing for 1 h at room temperature. Ni-NTA Superflow resin beads (17 g; Quiagen Redwood City, CA, USA) were equilibrated in denaturing buffer (6 M guanidine HCl, 100 mM Na Phosphate, 10 mM Tris, pH 8.0) for 1 h at room temperature. Solubilized inclusion bodies were centrifuged at 7900 × *g* for 5 min and the supernatant was added to the equilibrated beads and incubated for 1 h with gentle inversion to allow protein binding. The beads were then loaded into a XK16 (GE Healthcare Life Sciences; Pittsburgh, PA, USA**;** length 200 mm) glass column. Using an Amersham AKTA Explorer FPLC running Unicorn software (5.31 version, GE Healthcare Life Sciences; Pittsburgh, PA, USA), the protein was refolded with a linear gradient from 100% denaturing buffer to 100% refolding buffer (100 mM Na phosphate, 10 mM Tris, pH 8.0) over 4 h. Subsequently, the column was washed for 30 min with refolding buffer. The protein was then eluted from the column using a linear gradient from 100% refolding buffer to 100% elution buffer (500 mM imidazole, 100 mM Na phosphate, 10 mM Tris, pH 5.8). The central portion of the A_280_ UV peak was collected into dialysis buffer (10 mM Na Phosphate buffer, pH 5.8) to give ~4:1 ratio of eluted volume:dialysis buffer to complete pH conversion to 5.8. The purified protein was then filtered with a 0.22 μm Argos syringe filter prewashed with dialysis buffer, transferred into snakeskin dialysis tubing (MW cutoff 7 kDa; Pierce, Rockford, IL, USA) and placed in a 4 L beaker of dialysis buffer overnight at 4 °C with continuous stirring. Following dialysis, the protein solution was filtered again with a prewashed 0.22 μm Argos syringe filter, the concentration measured by 280 nm absorbance and 1 mL aliquots of the recombinant PrP^Sen^ batch were frozen in screw capped tubes at −80 °C. Each rPrP^Sen^ batch was obtained from a ~4 g cell pellet and yielded 10–15 mg of protein depending on the protein construct. All batches were stored at a concentration between 0.3 and 0.7 mg/mL.

### 2.2. RT-QuIC

Ninety-eight microliters of a reaction mixture containing a final concentration of 10 mM phosphate buffer (pH 7.4), 130–500 mM NaCl (as indicated), 0.1 mg/mL rPrP^Sen^, 10 μM ThT (Sigma, St. Louis, MO, USA; T3516-25G), and 1 mM ethylenediaminetetraacetic acid tetrasodium salt (EDTA) was loaded into each well of a black-walled 96-well plate with a clear bottom (Nunc, Rockford, IL, USA; 265301) and reactions were seeded with 2 μL of the indicated test dilution for a final reaction volume of 100 μL. All reactions contained a final concentration of 0.001%–0.003% SDS (Sigma; L6026-250G) as indicated. Reactions were incubated at 42–60 °C and shaken every other minute at 700–1100 revolutions per minute (rpm), as indicate. Brain homogenate serial dilutions were done using 0.5 mL and 1.5 mL Fisherbrand screw cap tubes (Fisherbrand, Rockford, IL, USA; catalogue number 02-681-334 and 02-681-339, respectively). Plates were sealed (Nalgene Nunc International sealer; 232702) and incubated in a BMG Fluostar plate reader (BMG Labtech, Cary, NC, USA) or a ThermoFisher iEMS Incubator/Shaker HT (Rockford, IL, USA) multiplate incubator/shaker as indicated, for 45–90 h with cycles of 60 s shaking and 60 s of rest throughout the incubation, unless otherwise indicated. ThT fluorescence measurements (450 ± 10 nm excitation and 480 ± 10 nm emission; bottom read) were taken every 45 min when incubating in the BMG Platereader. Alternatively, ThT readings were taken prior to incubation with the multiplate incubator/shaker, and then again after 17 and 22 h of incubation. In the latter case, the plates were moved directly to a BMG Fluostar plate reader for fluorescence measurements. Fluorescence reactions were judged to be positive or negative as described previously [16].

RT-QuIC data were analyzed as previously described [16]. Briefly, to compensate for differences between the plate readers, we averaged data from replicate wells and normalized to a percentage of the maximal fluorescence response of the instrument. The obtained values were plotted against the reaction times.

## 3. Results

### 3.1. Comparison of RT-QuIC Kinetics Using Multiple Batches of a Given Type of Recombinant PrP Substrate

**Figure 1:**
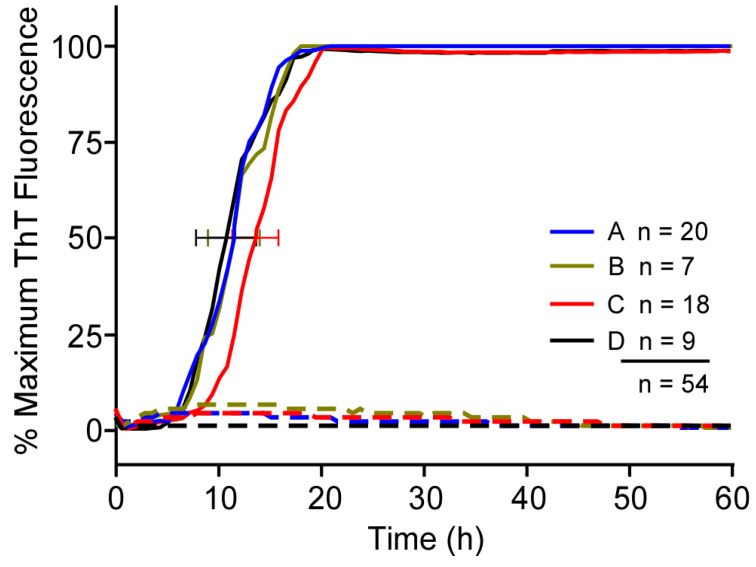
Real-time quaking-induced conversion (RT-QuIC) using four different hamster (Ha) recombinant PrP^Sen^ (rPrP^Sen^) substrate batches over approximately one year. Traces represent the average thioflavin-T (ThT) fluorescence from multiple experiments (n) using an individual Ha rPrP^Sen^ 90–231 substrate (Batch A–D). Each quadruplicate RT-QuIC reaction was seeded with 2 μL of a 5 × 10^−7^ dilution of a single hamster scrapie brain homogenate (263K strain) containing ~100 fg of PrP^Res^ (continuous lines) or a single hamster normal brain homogenate (dotted lines). Error bars denote standard deviation (SD) of time to half maximum ThT fluorescence calculated independently for each Ha 90–231 rPrP^Sen^ batch. All 54 independent experiments were performed as previously described at 42 °C [3]. The increase of the average normalized ThT fluorescence of replicate wells is plotted as a function of time.

To evaluate the consistency of our previously established RT-QuIC reactions, we performed a retrospective analysis of 54 independent experiments set up by the same operator over a year’s time (Figure 1). Recombinant PrP^Sen^ batches were utilized following storage of single use aliquots at −80 °C for 2–385 days. Reactions using four different Syrian hamster (Ha) rPrP^Sen^ (90–231) substrate batches were seeded with 5 × 10^−7^ tissue dilutions of a single hamster scrapie (263K strain) brain homogenate (ScBH) or a normal hamster brain homogenate (HaNBH). Multiple plate readers and at least six separate preparations of RT-QuIC stock solutions for a final reaction mixture composed of 10 mM phosphate buffer (pH 7.4), 300 mM NaCl, 0.1 mg/mL rPrP^Sen^, 10 μM ThT and 1 mM EDTA, were used. All of the reactions gave strongly positive enhancements in ThT fluorescence with a ~5 h standard deviation in the time to half maximal fluorescence for each substrate batch and an overall range of ~10 h across all batches. Thus, each of the batches was responsive in multiple RT-QuIC experiments, albeit with some variability in the RT-QuIC kinetics, whether measured by the time to half-maximal fluorescence or the lag phase (the point in time where a positive ThT fluorescence signal is first detected relative to the pre-transition base line).

### 3.2. Higher Temperatures Promote Faster RT-QuIC Reactions

**Figure 2:**
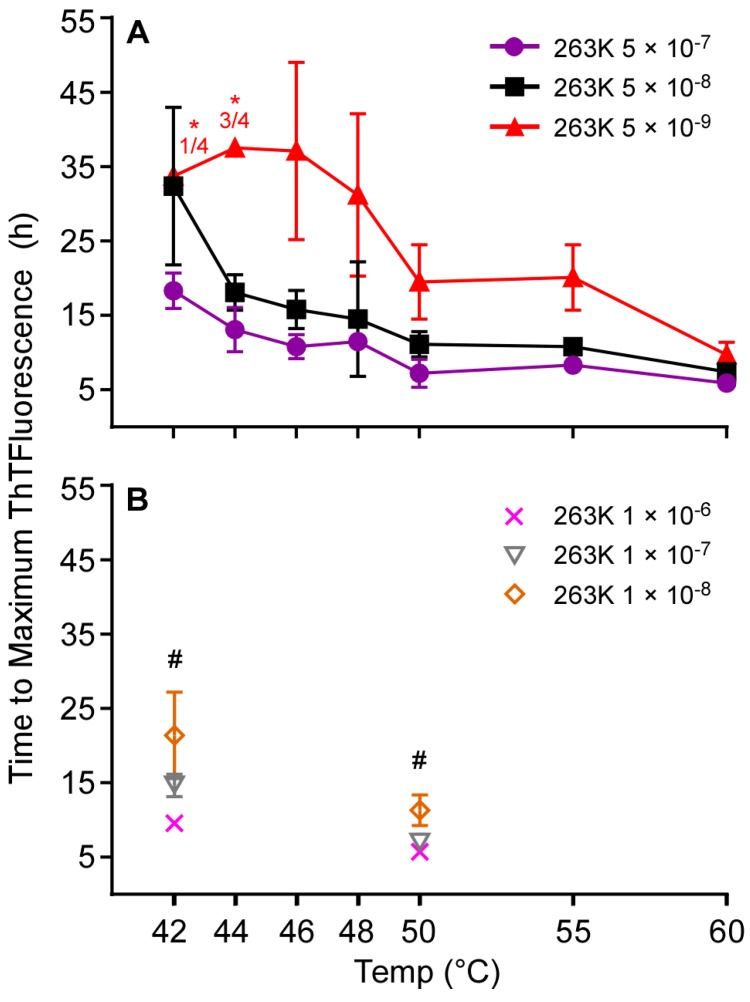
Effect of temperature elevation on RT-QuIC detection of hamster 263K brain homogenate derived seeding activity using Ha rPrP^Sen^ 90–231. RT-QuIC reactions seeded with the designated hamster 263K positive brain tissue dilutions were incubated at temperatures of 42–60 °C (*x* axis). The graphs (A, B) show the average time (hours, *y*-axis) to maximum ThT fluorescence (±SD) of quadruplicate reactions for each brain tissue dilution. * Fractions in red (A) indicate the number of ThT positive replicate wells (numerator) out of the set of four replicate wells (denominator). No SD was calculated for the reactions marked with an asterisk (*). (#) Differences were statistically significant (B) with *p* values < 0.002, 0.001 and 0.05 for the 1 × 10^−6^ (X), 1 × 10^−7^ (▽) and 1 × 10^−8^ (◊) 263K brain tissue dilutions, respectively.

**Figure 3:**
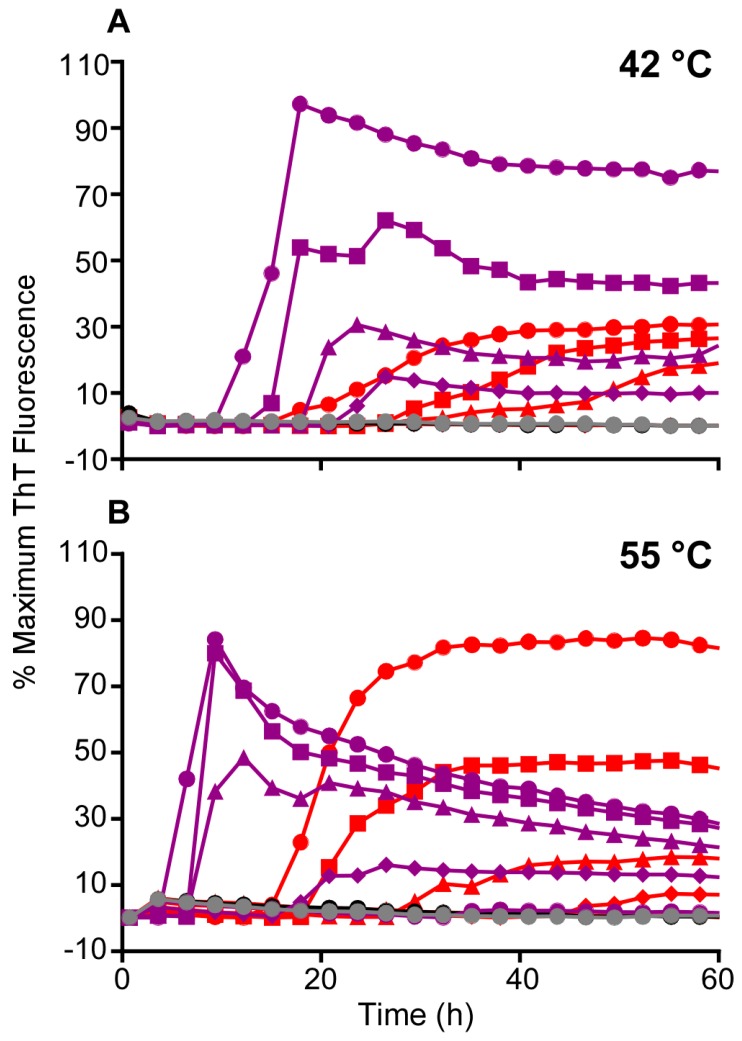
Comparison of RT-QuIC detection of hamster 263K brain homogenate derived seeding activity using Ha 23–231 or 90–231 at 42 and 55 °C. Quadruplicate RT-QuIC reactions were seeded with serial dilutions of 5 × 10^−7^ (●), 5 × 10^−8^ (■), 5 × 10^−9^ (▲) and 5 × 10^−10^ (♦) of hamster 263K scrapie strain positive brain homogenate. Negative control reactions were seeded with a 5 × 10^−7^ dilution of hamster normal brain homogenate (black and gray traces for Ha 23–231 and Ha 90–231 rPrPSen, respectively). The reactions were incubated at either 42 °C (A) or 55 °C (B) using Ha 23–231 (red) or 90–231 (purple) rPrPSen as substrate. The increase of the average normalized ThT fluorescence of replicate wells is plotted as a function of time. Although ThT readings were taken every 45 min, for visual clarity, the curves report only readings at 135-min intervals. We have observed similar enhancements of the speed of the reactions in two additional experiments.

Building on our previous observations that the use of Ha rPrP^Sen^ (90–231) substrate and a 55 °C reaction temperature improved the speed and sensitivity of RT-QuIC detection of sCJD seeds in CSF [12], we systematically investigated the effect of increasing the RT-QuIC reaction temperature. In RT-QuIC reactions using this substrate and a range of ScBH seed dilutions (5 × 10^−7^–5 × 10^−9^), we observed 2–3-fold reductions in the time to reach maximum ThT fluorescence by increasing temperature from 42 to 60 °C at three extreme dilutions of brain homogenate (BH) near the detection limit of the assay (Figure 2). At temperatures up to 60 °C none of the negative control reactions seeded with HaNBH were positive within 50 h (data not shown). Comparison of results from three independent experiments performed at 42 °C and 50 °C using 10^−6^–10^−8^ dilutions of ScBH confirmed that the higher temperature gave shorter times to maximum ThT fluorescence at each seed dilution (Figure 2B). In a further comparison of 55 °C *versus* 42 °C (Figure 3A-B), comparable detection was observed with a serial dilution analyses of ScBH, using Ha (23–231; Figure 3) or Ha (90–231; Figure 3) rPrP^Sen^ substrates. That is, with both temperatures the most extreme dilution eliciting positive reactions was 5 × 10^−10^. Furthermore, we observed an overall faster decrease of the ThT signal at higher temperatures (Figure 3B) which occurred after the reactions reached maximum ThT fluorescence. Overall, we conclude that when using Ha (23–231) or Ha (90–231) rPrP^Sen^, higher temperatures increased the speed of ScBH-seeded RT-QuIC reactions with comparable detection of seeding activity in samples as dilute as 5 × 10^−10^ Sc brain tissue dilution.

### 3.3. Improved Detection of Human sCJD in Nasal Brushings

**Figure 4:**
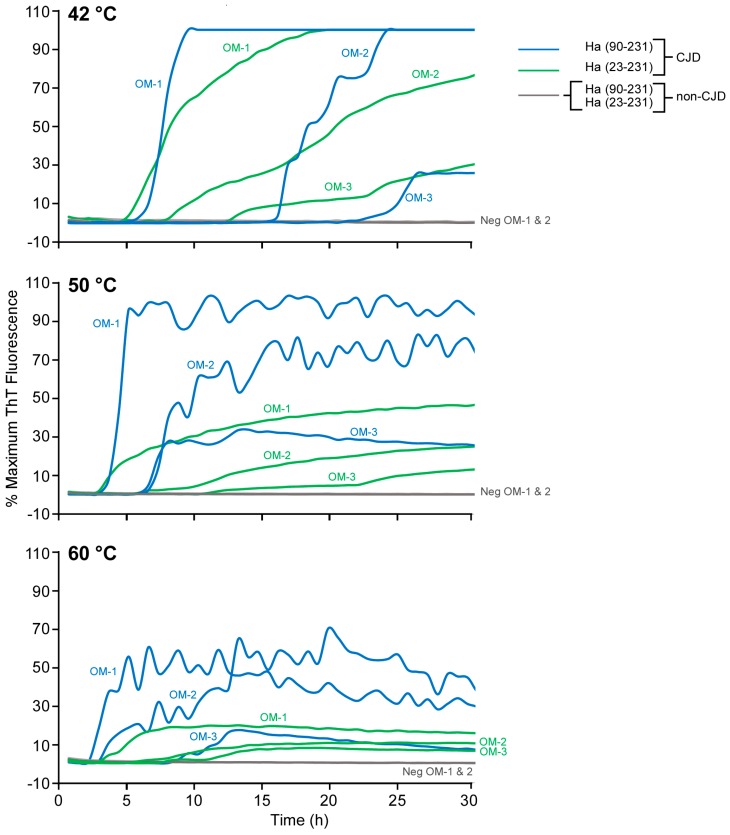
RT-QuIC detection of seeding activity in olfactory mucosa samples (OM) from human sCJD patients using Ha 23–231 or Ha 90–231 rPrP^Sen^ substrates at 42, 50 and 60 °C. Olfactory mucosa samples from three sCJD (OM 1, 2 and 3) and two non-CJD patients (Neg OM 1 and 2) serially diluted to 4 × 10^−5^ tissue dilutions were used to seed quadruplicate reactions with either Ha 90–231 (sCJD blue lines; uninfected gray) or Ha 23–231 (sCJD green lines; uninfected gray) rPrP^Sen^ as substrates. Replicate reactions were incubated at the indicated temperatures. The increase of the average normalized ThT fluorescence of replicate wells (*y*-axis) is plotted as a function of time (*x*-axis).

Nasal olfactory mucosa (OM) brushings have provided the basis for the highly sensitive and specific diagnosis of CJD when analyzed at 42 °C using Ha rPrP^Sen^ (23–231) substrate [16,17]. Considering the above results in Figure 2 and Figure 3, we attempted to improve detection of sCJD seeds in OM brushings [16] by raising the temperature and using Ha rPrP^Sen^ (90–231) as substrate. Figure 4 shows that at 50 °C and 60 °C Ha rPrP^Sen^ (90–231) gave shorter lag phases with higher maximum ThT fluorescence levels in reactions seeded with dilutions of OM samples from three different pathologically confirmed definite sCJD patients, but not CJD-negative controls. We also observed a reduced maximum fluorescence from most of the sCJD OM-seeded reactions with increased variability of the mean fluorescence intensity at the higher temperatures. Nonetheless, overall these results suggest that the speed of RT-QuIC detection for sCJD OM specimens is increased by raising the reaction temperature and the signal strength is increased by using the Ha rPrP^Sen^ (90–231) substrate.

### 3.4. Longer Shaking Intervals Promote Faster RT-QuIC Kinetics

**Figure 5:**
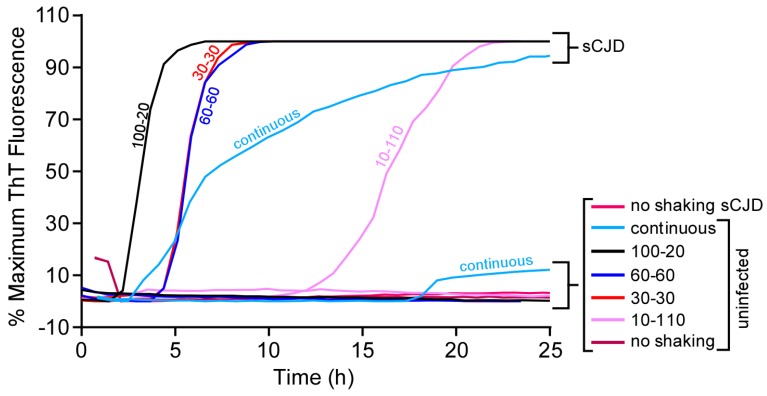
Effect of shaking conditions on RT-QuIC amplification kinetics. Reactions were seeded with either 2 μL of 1 × 10^−7^ sCJD or Alzheimer diseased brain tissue dilutions and incubated at 42 °C. Each trace represents the averaged normalized % maximum ThT signal (*y*-axis) of four replicate wells plotted as a function of time (*x*-axis). Human rPrP^Sen^ (23–231) was used as the substrate with a final NaCl concentration of 130 mM. The reactions were either continuously shaken (light blue), or subjected to cycles of shake and rest of variable duration (100, 60, 30, 10 s shake in combination with 20, 60, 30 or 110 s rest, black, royal blue, bright red and purple, respectively). Reactions that were not subjected to any shaking (dark red) were also examined.

RT-QuIC has involved double orbital or orbital shaking to promote seeded polymerization of rPrP^Sen^ [3,4,13]. To further characterize the influence of double orbital shake-rest cycle variations on RT-QuIC kinetics we used the BMG Fluostar plate reader and compared cycles with equal periods of shake and rest (either 30 or 60 s) *versus* unequal shake and rest periods, continuous shaking, and no shaking. Here, we used human rPrP^Sen^ (23–230) as a substrate and seeded reactions with either 1 × 10^−7^ sCJD or Alzheimer’s disease brain tissue dilution. When sCJD-seeded reactions were incubated at 42 °C without shaking there was no ThT fluorescence enhancement within 24 h (Figure 5). A 10 s shake — 110 s rest cycle decreased the lag phase to ~15 h, while 30 s shake—30 s rest and 60 s shake—60 s rest cycles further shortened the lag phase to ~5 h (Figure 5). The shortest lag phases (~3 h) were seen with the 100 s shake—20 s rest cycle, although a higher overall incidence of prion-independent conversion of the rPrP^Sen^ substrate at ~15 h was also observed with continuous shaking (Figure 5). Similar trends were seen when RT-QuIC reactions were seeded with the same serial dilutions of sCJD BH using either the Ha rPrP^Sen^ (23–231) or Ha rPrP^Sen^ (90–231) substrates (data not shown). These results indicated that continuous shaking reduced prion-specificity. However, faster RT-QuIC reactions could be achieved without apparent specificity loss by increasing the shake-rest ratio, while maintaining a short rest period within a 2-min cycle at 42 °C.

### 3.5. Acceleration of RT-QuIC with Higher Shaking Speeds

**Figure 6:**
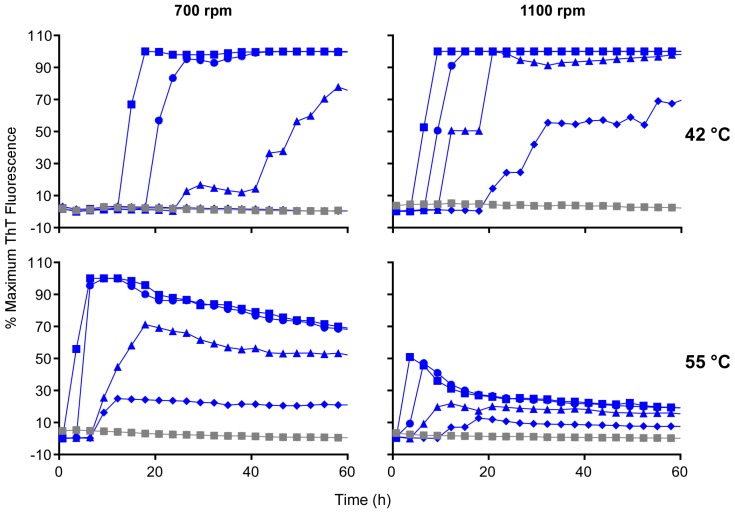
Effects of temperature in combination with different double orbital shaking intensities on RT-QuIC sensitivity. Quadruplicate RT-QuIC reactions were seeded with 2 μL of scrapie positive (263K) brain tissue dilutions of 5 × 10^−7^ (■), 5 × 10^−8^ (●), 5 × 10^−9^ (▲), and 5 × 10^−10^ (♦) or uninfected hamster brain tissue at a dilution of 5 × 10^−7^ (gray) using N-terminally truncated (residues 90–231) Ha rPrP^Sen^ as substrate. Each trace represents the average ThT signal of quadruplicate wells seeded with the same indicated brain homogenate dilution. The reactions were incubate at 42 °C or 55 °C and subjected to 700 or 1100 rpm double orbital shaking, as indicated. Although ThT readings were taken every 45 min, for visual clarity, the curves report only readings at 135-min intervals.

To further investigate the effect of mechanical energy of shaking on the RT-QuIC, we also evaluated whether higher double orbital (*i.e.*, figure eight shaped) shaking speeds using the BMG Fluostar plate reader could improve RT-QuIC performance. We used Ha rPrP^Sen^ (90–231) as the substrate in reactions seeded with 10-fold serial dilutions of ScBH from 5 × 10^−7^ to 5 × 10^−10^. Increasing the shaking speed from 700 to 1100 rpm shortened the lag phases to an extent comparable to that observed by increasing the temperature from 42 °C to 55 °C at 700 rpm (Figure 6). However, increasing both the temperature and the shaking speed at the same time did not always result in further shortening of the lag phases. Our direct comparison using the same batch of Ha rPrP^Sen^ (90–231) indicates that increasing the shaking speed alone also allowed more consistent detection of seeding activity in reactions seeded with the highest brain tissue dilutions (*i.e.*, 5 × 10^−10^, Figure 6). Overall our results suggest that RT-QuIC kinetics can be accelerated by either changing the duration or the intensity of shaking at a set temperature.

### 3.6. Effect of pH on RT-QuIC Amplification Kinetics

**Figure 7:**
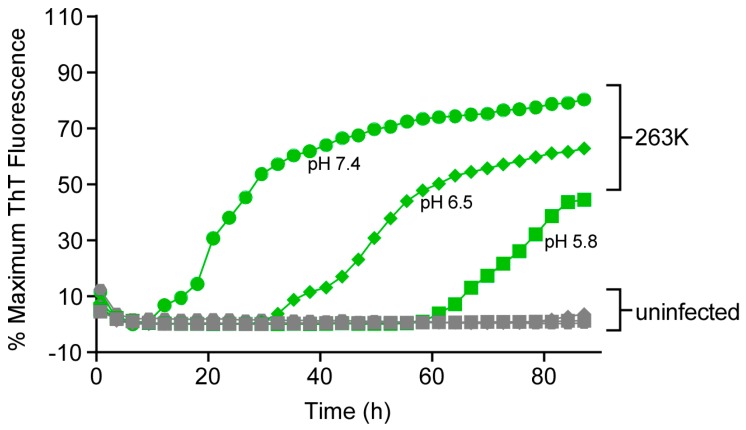
Influence of pH on RT-QuIC amplification kinetics. Quadruplicate RT-QuIC reactions were seeded with 2 μL of 5 × 10^−8^ scrapie positive (263K, green) or uninfected (gray) hamster brain tissue dilutions. The reaction mix was prepared as indicate in Materials and Methods with adjustments of the final pH of to 7.4, 6.5 or 5.8 as indicated. The samples were incubated at 42 °C and Ha rPrP^Sen^ 23–231 was used as the substrate. Several independent experiments performed under the same conditions but at an even lower pH of 4.5, showed elongated lag phases (greater than 90 h), confirming our observations. Although ThT readings were taken every 45 min, for visual clarity, the curves report only readings at 135-min intervals.

Previous studies have reported that, under somewhat different RT-QuIC conditions, lower pHs can aid sensitive detection of human PrP^sCJD^ in brain and CSF samples [4,24]. Within the same experiment, we compared pH 6.5 and 5.8, as alternatives to our standard of 7.4, for detection of prion seeding activity under our experimental conditions using the ScBH seed with Ha rPrP^Sen^ (23–231) substrate (Figure 7). With lower pH the reactions had elongated lag phases. Overall, these results indicated that under our current conditions, at least with this seed and substrate, pH 7.4 was preferable.

### 3.7. Influence of SDS on RT-QuIC Amplification Kinetics

**Figure 8:**
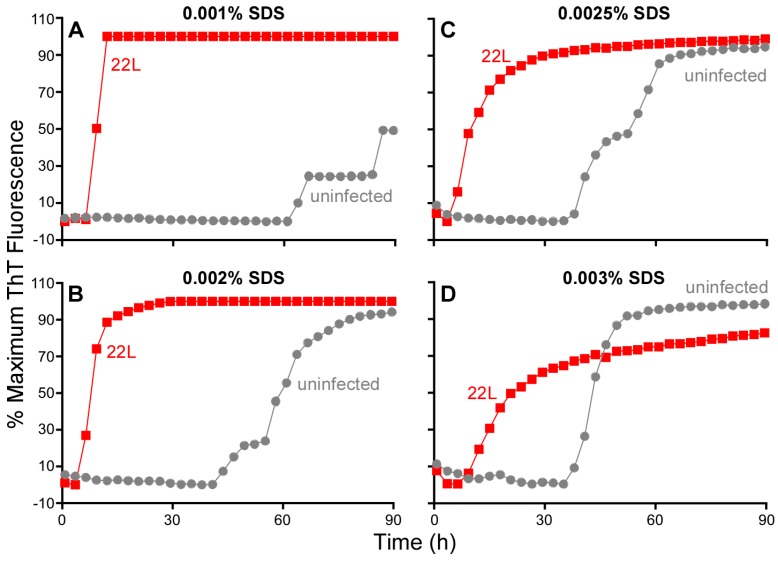
Effect of SDS on prion seeded and unseeded RT-QuIC reactions. Brain homogenate from 22L prion strain infected (red) and uninfected (gray) mouse were serially diluted in 0.1% SDS/PBS/N2 solution. The final dilution (1 × 10^−4^ brain tissue dilution) used to seed each well was prepared to give a final SDS concentration per reaction of: 0.001% (**A**); 0.002% (**B**); 0.0025% (**C**); or 0.003% (**D**). Mouse rPrP^Sen^ (23–231) was used as the substrate in combination with 130 mM NaCl and all conditions were run in a single reaction plate. The average normalized fluorescence (*y*-axis) from replicate wells is plotted as a function of time (*x*-axis, h). Although ThT readings were taken every 45 min, for visual clarity, the curves report only readings at 135-min intervals.

**Figure 9:**
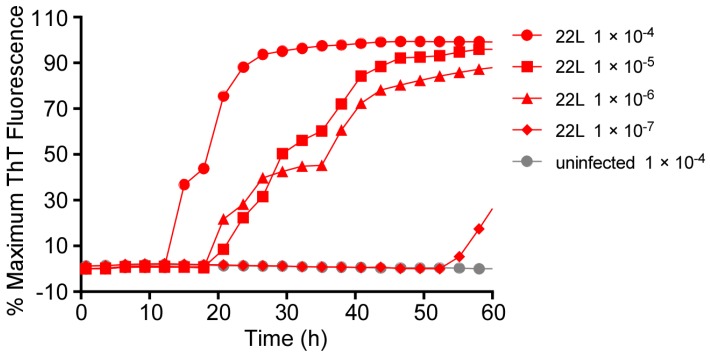
Sensitivity of the RT-QuIC for the detection of 22L prion seeding activity in the presence of 0.001% SDS. Brain homogenate from a 22L prion strain infected (red) and an uninfected (gray) mouse were serially diluted in 0.1% SDS/PBS/N2 solution. The final indicated dilutions were prepared to give a final SDS concentration per reaction of 0.001%. The substrate was Mouse rPrP^Sen^ (23–231) in combination with 130 mM NaCl. The average normalized fluorescence from replicate wells is plotted as a function of time. Although ThT readings were taken every 45 min, for visual clarity, the curves report only readings at 135-min intervals.

Thus far, we have described RT-QuIC testing of either BH or OM samples in which tissues were diluted in a buffer containing a final SDS concentration of 0.002%. In other studies, we have found that the inclusion of 0.002% SDS with human CSF-seeded RT-QuIC reactions decreased responsiveness to sCJD seed with the Ha rPrP^Sen^ (23–231) substrate but markedly increased the speed and strength of sCJD detection using Ha rPrP^Sen^ (90–231) [12]. We also reported detection of prion seeding activity from 28 types of TSEs by RT-QuIC using Bank Vole 23–230 M_109_ in combination with a final concentration of 0.001% SDS [21]. To further explore effects of SDS on RT-QuIC reactions, we varied the SDS concentrations in RT-QuIC reactions seeded with mouse scrapie brain homogenates (22L or RML strains) using mouse (Mo) rPrP^Sen^ (23–230) substrate (Figure 8). We were particularly interested in this substrate not only because of its utility for detecting mouse-adapted scrapie prions [6] but also because of problems that we had encountered with early (~40 h) prion-independent (NBH-seeded) false-positive responses with several recent batches of this substrate (e.g., Figure 8B). In NBH seeded reactions, reducing the SDS concentration by half (from 0.002% to 0.001%) delayed prion-independent false-positive responses markedly while still allowing for rapid seeding by 10^−4^ dilutions of 22L ScBH (Figure 8A). On the other hand, increasing the SDS concentration by just 1.25 to 1.5 fold accelerated the false-positive responses and slowed the scrapie-seeded responses (Figure 8C,D). Additional experiments with serial dilutions of 22L ScBH and NBH confirmed that reducing the final SDS concentration from 0.002% to 0.001% significantly delayed false positive (NBH-seeded) reactions, while also increasing the proportional distinction in lag phase between the 22L ScBH (down to 10^−6^ dilution)—and NBH-seeded reactions (Figure 9). Collectively, these findings emphasize that small changes in SDS concentration can have profound effects on RT-QuIC reaction kinetics, specificity and sensitivity that are dependent upon substrate and sample type.

### 3.8. Prion Seed Detection Using a Separate Shaker-Incubator and Single-Timepoint Readings on a Fluorimeter

**Figure 10:**
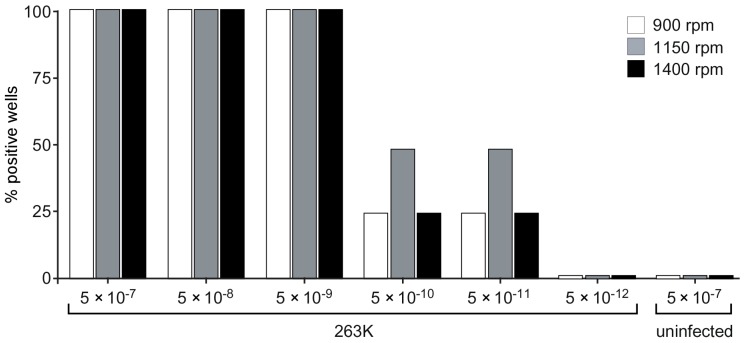
Effect of different orbital shaking conditions (900, 1150 or 1400 rpm) on RT-QuIC sensitivity using an iEMS multi-plate, temperature-controlled shaker incubator. Quadruplicate RT-QuIC reactions were seeded with 2 μL of scrapie positive (263K) brain tissue dilutions as indicated. Reactions were incubated at 42 °C in an orbital shaking Thermo Scientific (iEMS Incubator/Shaker HT) using Ha rPrP^Sen^ (90–231) as the substrate. After a 17 h incubation, ThT fluorescence was measured in a BMG FLUOstar Omega plate reader using the RT-QuIC read settings described in Materials and Methods. The percentage of positive replicate wells is plotted for each dilution used.

Although the ability to monitor the progression of RT-QuIC over time can be helpful in assay development, it requires that a single multiwell plate occupies a shaking, temperature controlled fluorescence plate reader for the entire duration of the test. Once reaction parameters are established for a given sample type, it is likely that fluorescence readings at a single time point would suffice to discriminate prion-positive and -negative samples. Such established reactions could then be performed more economically if multiple plates were shaken in a temperature-controlled plate shaker without fluorimetry optics and then removed at a specific time point for endpoint readings in a simple fluorescence plate reader (without shaking or temperature control mechanisms). To test this possibility, we used a multi-plate shaking incubator (Scientific, iEMS Incubator/Shaker HT). Because the orbital shaking motion of the iEMS incubator-shaker differs from that of our double-orbital shaking fluorescence plate readers, we tested the available orbital shaking speed options (900, 1150 and 1400 rpm). Reactions seeded with serial dilutions of ScBH were incubated at 42 °C using Ha rPrP^Sen^ (90–231) as the substrate. When shaken at 900–1400 rpm, we observed positive reactions in at least one of four replicate wells within a 17 h reaction with ScBH dilutions down to 5 × 10^−11^ (Figure 10), but not in control reactions seeded with HaNBH. At 22 h, spontaneous conversion of the substrate in HaNBH seeded reactions was observed suggesting that the reaction time point for fluorescence readings should be selected carefully to reduce the possibility of false positive reactions. Nonetheless, collectively, these findings indicate that at empirically determined shaking speeds, dependent upon the apparatus in question, a single endpoint fluorescence readings in a separate fluorimeter, can allow the specific detection of prion seeding activity by RT-QuIC with little, if any, loss of sensitivity.

## 4. Discussion

To facilitate optimization of RT-QuIC applications to a wider variety of samples, we have evaluated key reaction parameters that can improve the speed, sensitivity, throughput and cost of these tests (Table 1). Our comparisons of Ha rPrP^Sen^ (23–231) and (90–231) as substrates in ScBH seeded reactions indicated that although both substrates provide similar sensitivities, rPrP^Sen^ (90–231) gave shorter lag phases at each scrapie seed concentration. Raising the temperature increased the reaction speed for both substrates, while increasing the double orbital shaking rpm increased both the speed and sensitivity of ScBH-seeded reactions using the rPrP^Sen^ (90–231) substrate. Faster reaction rates (so far) have been achieved either by raising the temperature to 55 °C or by increasing the double orbital shaking speed to 1100 rpm. However, raising both the temperature and shaking speed to these levels simultaneously did not show additive beneficial effects. We currently recommend 55 °C because at 60 °C the incidence of false positives increases and fluorescence intensity is considerably diminished. A potential problem with higher shaking speeds and durations in the shake-rest cycles might be increased wear on instrumentation. However, this additional wear should be mitigated on a per experiment basis by shorter overall reaction times. In any case, we show that with sCJD and rodent adapted-scrapie brain seeds, judicious selection of variables including substrate, temperature and shaking intensity can markedly increase the speed and, often the sensitivity of RT-QuIC reactions.

As noted above (Figure 1), we have quantified the variation in RT-QuIC lag phases with different batches of rPrP^Sen^ (90–231). Interestingly, with one batch used in a previous publication [3], we achieved reaction speeds as good as the best reported here without using elevated temperatures (above 42 °C) or shaking intensities, and we do not know if raising temperature or shaking speed would have improved the performance of that long-gone batch. However, most of the rPrP^Sen^ (90–231) batches (>25) that we have used have given the typical RT-QuIC kinetics as seen in Figure 1. Controlled experiments with individual batches have demonstrated the strong beneficial effects of temperature and shaking intensity on RT-QuIC lag phases. In the development of new RT-QuIC applications for different sample types, it may not be safe to assume that conditions that are optimal for the seeds and substrates studied here will be the same for all other scenarios. Thus, these key variables should be optimized for new sample types and substrates.

We have previously described analysis of a variety of RT-QuIC conversion products by PK digestion and Western blot analysis [3,6,13,21,25,26,27]. Recovery of the RT-QuIC product from the 96-well plate requires vigorous scraping of the wells because of the adherence of the conversion product to the plate. We noted that recovery of these conversion products could be improved with the addition of CHAPS detergent at an empirically determined concentration (0.01%). Our further findings suggest that, where appropriate, inclusion of other components in the RT-QuIC, such as CHAPS, can help with RT-QuIC product recovery.

While the biochemical underpinnings of the effects that we have observed remain unclear, we can speculate as to some potential mechanisms. With respect to the faster RT-QuIC reaction kinetics using the Ha rPrP^Sen^ (90–231) substrate, it is possible that the lack of the flexible N-terminal residues 23–89 destabilizes the native PrP^Sen^ conformation, allowing it to more rapidly refold into the amyloid conformation under the influence of a prion seed. Alternatively, the N-terminal truncation might reduce the tendency of the substrate molecules to bind non-specifically to the reaction vessel or assume other off-pathway states, such as certain oligomers [28]. Temperature increases may also help to destabilize the Ha rPrP^Sen^ substrate, making it more prone to seeded conversion. Additionally, higher incubation temperatures or more vigorous shaking conditions might also increase the frequency of molecular collisions between seed and substrate to accelerate polymerization. In any case, both prion-seeded and prion-independent reactions were accelerated by higher temperatures and stronger, longer shaking, but the kinetic discrimination between prion-positive and negative samples was maintained within specific ranges. These mechanical, thermal and chemical ranges must provide an environment in which rPrP remains in solution and available as a substrate throughout the duration of the experiment, while remaining permissive to templating.

The reduced maximum fluorescence we often observed with higher incubation temperatures might have been due either to heat-induced effects on ThT’s association with the rPrP amyloid fibrils, or to effects on the fibrils themselves. For instance, a higher level of fibril bundling or adsorption to the surface of the well presumably would reduce the fibrillar surface area with which ThT interacts reducing fluorescence yield. Such an effect has been described for other amyloid fibrils [29]. The fact that the ThT readings leveled off in many reactions suggests that it is not heat-induced ThT degradation that accounts for all of the lower fluorescence. Despite the reduced ThT signals, positive signals were easily distinguished from negative control baselines.

Recent studies have described an alternative quantitative RT-QuIC approach that relies on comparisons of reaction lag phases [10,11]. We agree that this is a useful alternative strategy to end-point dilution quantitation [3]. However, the lag phase variability that we observed in over 50 independent experiments (Figure 1), and the likelihood that variance would continue to increase with increasingly weaker seeding strengths, suggests that lag phase-based quantitation should be deployed with appropriate standard curves and careful matching of test sample type and preparation, rPrP^Sen^ substrate, and buffer constituents within each experiment, preferably performed on the same plate. A thorough examination of the implications of the variance in reaction kinetics with prion seed titer has been reported recently by others [30].

In most applications of the RT-QuIC assay, enhanced throughput and reduced cost will be advantageous. The use of a multi-plate incubator-shaker allows multiple 96-well plates to be run simultaneously in a device that cost much less than a single-plate shaking- and temperature-controlled fluorescence plate reader. Using such a system, a laboratory might need only a single fluorescence plate reader (without shaking or temperature control) to read plates run on multiple instruments, allowing for much higher throughput RT-QuIC testing per unit cost for instrumentation. Although this scenario would limit resolution of differences in lag phase between samples, it should be adequate for circumstances in which assessments are needed at only a limited number of time points.

In conclusion, our findings further support the broad applicability of the RT-QuIC and identify measures that can be optimized to substantially improve the speed, sensitivity, throughput and cost-effectiveness of RT-QuIC assays.

## Figures and Tables

**Table 1:** Currently implemented RT-QuIC conditions *.

Species	Accession Number	Amino Acid Residues	Genotype	SDS (%)	NaCl (mM)	Temperature (°C)	Prion Types Detected	References
**Hamster**	K02234	23–231	NA	0.002	300, 324	42	263K, sCJD, vCJD, L-BSE	[3,9,19]
		90–231	NA	0.002	300, 350	42, 55	263K, sCJD, CWD, HY-TME, L-BSE	[3,8,9,12,19,20]
**Hamster-Sheep Chimera**	Hamster K02234	Hamster 23–137	NA	0.002	300	42	vCJD, C-BSE, L-BSE	[5,19]
	Sheep AY907689	Sheep 141–234	NA					
**Bank vole**	AF367624	23–230	M109	0.001	300	42	sCJD, vCJD, iCJD, GSS, gCJD, FFI, sFI, 6-octarepeat insert, CS, AS, C-, L- and H-type BSE, CWD, 263K, HY- and DW-TME, Chandler, ME7, 87V, 22L WT and GPI^−^	[21,22]
		90–230	M109	0.001	300	55	H-BSE, L-BSE	[22]
		23–230	I109	0.001	300	42	H-BSE, L-BSE	[22]
**Mouse**	M13685	23–231	NA	0.001	130	42	RML, RML GPI^−^, ME7, ME7 GPI^−^, 22L, 22L GPI^−^, C-BSE, H-BSE, L-BSE	([6] Figure 9 and Figure 10 **), [22]
**Sheep**	AJ567988	25–234	VRQ	0.002	200, 300, 400	42	Sheep scrapie (VRQ), goat scrapie	[3,23]
	AY907689	25–234	ARQ	0.002	200	42	Goat scrapie	[23]
	HM639748	25–234	ARR	0.001	300	42	H-BSE, L-BSE	[22]
**Human**	M13899	23–231	M129	0.002	130	42	sCJD, vCJD, H-BSE, L-BSE	[9,19,22]
		23–231	V129	0.002	130	42	sCJD, vCJD	[9]
**Human-Bank vole Chimera**	Human M13899	Human 23–165	M129	0.001	300	42	H-BSE, L-BSE	[22]
	Bank vole AF367624	Bank vole 166–230	NA					
**Deer**	AF156185	24–234	GMSSQ	0.002	300	42	CWD	[3]

Abbreviations: Creutzfeldt-Jakob disease (sCJD), Variant Creutzfeldt-Jakob disease (vCJD), Iatrogenic Creutzfeldt-Jakob disease (iCJD), Gerstmann-Sträussler-Scheinker disease (GSS), Genetic Creutzfeldt-Jakob disease (gCJD), Fatal Familial Insomnia (FFI), Sporadic Familial Insomnia (sFI), Classical Scrapie (CS), Atypical Scrapie (AS), Classical (C-), Atypical L- and H-type bovine spongiform encephalopathies (BSE), Chronic Wasting disease (CWD), Hyper (HY) and Drowsy (DW) Transmissible Mink Encephalopathy (TME), wild type (WT) and non-glycosylphosphatidylinositol (GPI)-anchored PrP (GPI^−^), Deer PrP genotype G_96_M_132_S_138_S_225_Q_226_ (GMSSQ), Sheep PrP genotype V_136_R_154_Q_171_ (VRQ), Sheep PrP genotype A_136_R_154_Q_171_ (ARQ), Sheep PrP genotype A_136_R_154_R_171_ (ARR); * RT-QuIC conditions currently used by the authors, the references list a selection of papers in which ranges of such conditions have been tested; ** Figures referenced from the current publication.
